# Research Advances on the Interactions between Rabies Virus Structural Proteins and Host Target Cells: Accrued Knowledge from the Application of Reverse Genetics Systems

**DOI:** 10.3390/v13112288

**Published:** 2021-11-16

**Authors:** Juanbin Yin, Xiangwei Wang, Ruoqing Mao, Zhixiong Zhang, Xin Gao, Yingying Luo, Yuefeng Sun, Xiangping Yin

**Affiliations:** State Key Laboratory of Veterinary Etiological Biology, Lanzhou Veterinary Research Institute, Chinese Academy of Agricultural Sciences, Lanzhou 730046, China; yinjuanbin@163.com (J.Y.); wangxiangwei@caas.cn (X.W.); maoruoqing@caas.cn (R.M.); zhangzhixiong1006@163.com (Z.Z.); gaoxin1992@outlook.com (X.G.); yingyingluo0325@163.com (Y.L.)

**Keywords:** rabies virus, structural proteins, pathogenesis, reverse genetics systems, attenuated vaccine strains

## Abstract

Rabies is a lethal zoonotic disease caused by *lyssaviruses*, such as rabies virus (RABV), that results in nearly 100% mortality once clinical symptoms appear. There are no curable drugs available yet. RABV contains five structural proteins that play an important role in viral replication, transcription, infection, and immune escape mechanisms. In the past decade, progress has been made in research on the pathogenicity of RABV, which plays an important role in the creation of new recombinant RABV vaccines by reverse genetic manipulation. Here, we review the latest advances on the interaction between RABV proteins in the infected host and the applied development of rabies vaccines by using a fully operational RABV reverse genetics system. This article provides a background for more in-depth research on the pathogenic mechanism of RABV and the development of therapeutic drugs and new biologics.

## 1. Introduction

Rabies is a fatal zoonotic disease caused by *lyssaviruses*, such as rabies virus (RABV), which infects humans and other mammals [1]. The disease remains an important agricultural and public health problem worldwide. Every year, approximately 12 to 17 million people are bitten by rabid animals, predominantly by dogs. This translates to tens of thousands of human fatalities annually [2].

Taxonomically, RABV belongs to the genus *Lyssavirus*, family *Rhabdoviridae*. The etiological agents are enveloped, non-segmented, negative-stranded RNA virus with a total genome length of about 12 kb, encoding five structural proteins: the nucleoprotein (N), the phosphoprotein (P), the matrix protein (M), the glycoprotein (G), and the large RNA polymerase protein (L). Mature RABV particles are composed of a nucleocapsid (NC) and envelope. The NC is composed of the N, P, and L proteins. The NC forms a ribonucleoprotein (RNP) complex with the viral genome. The RNP plays an important role in viral replication and transcription. The G and M proteins form the envelope of the virus [3]. Among them, the G protein is capable of inducing the production of virus neutralizing antibodies (VNA), and mediates cellular tropism and absorption by neuronal cells [4,5]. These five structural proteins work together to regulate viral replication, transcription, infection, and evasion of the host immune response. The aim of this study is to summarize the interactions between RABV structural proteins and host cells and to review the progress in reverse genetics systems to better understand viral pathogenic mechanisms and to develop novel biologics [6,7,8,9,10].

## 2. Interactions between RABV Structural Proteins and Host Target Cells

**Figure 1:**
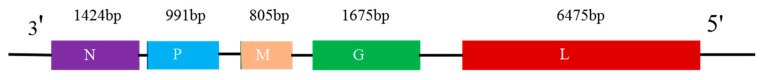
Genome structure of rabies virus.

The five RABV structural proteins, N, P, M, G, and L (Figure 1), play vital roles in assembling mature virus particles. Through electron microscopy, knoblike protuberances are visualized on the viral surface, and a series of honeycomb-like stripes are observed within the virion’s interior [11,12]. The G protein is the only one exposed on the virion’s surface. The M protein has been proposed to bridge the RNP and the cytoplasmic domain (CD) of the RABV G to form the bullet-shaped virion [13]. The N, P and L proteins combine tightly with the genomic RNA to form the RNP, which acts as the active center for viral transcription and replication [14].

### 2.1. The Pathogenic Mechanisms of RABV N Protein

The N protein participates in the packing or folding of viral particles and forms a tight RNP complex, together with the P and L proteins, which initiates RABV mRNA transcription and full-length genomic RNA replication. This N protein also encapsidates and protects the genomic RNA from cleavage by cellular nucleases [3]. In addition, the N protein plays an important role in evading innate immunity and modulates pathogenicity in the host [15]. Shimizu et al. [16] reported that changes in the N, P, and M genes (other than RABV G gene) altered the virulence of avirulent strains, indicating that these genes were related with the pathogenicity of RABV in adult mice. This work also demonstrated that two amino acid mutations at positions 394 and 395 in the N protein of the Ni CE strain (involved with the P protein binding domain) [17], may affect the transport of P protein in cells, thus affecting the ability of this protein to inhibit host antiviral responses. Mei et al. [18] recombined the N gene of the HEP-Flury strain, transferring it from 1 (N-P-M-G-L) to 2 (P-N-M-G-L), 3 (P-M-N-G-L), or 4 (P-M-G-N-L) of the wild-type virus. They found that the replication and transmission ability of the rHEP-Flury could be reduced by the N gene rearrangement in the genome. Masatani et al. [19,20,21] compared the ability of inhibiting the IFN response in the mouse brain by using cultured neuroblastoma cell lines with several RABV strains (e.g., the Nishigahara (Ni) strain; the attenuated virus derived Ni-CE and a strong chimeric virus CE (Ni-N) with a Ni-CE genetic background). This demonstrated that the Ni and CE (Ni-N) inhibited the induction of type I IFN. Studies have also shown that amino acids at 273 and 394 of the N protein (known as pathogenicity determinants) inhibit the production of IFN to affect virus entry in the brain [20]. Mutations at 273 and 394 sites of the Ni N protein effectively avoided RIG-I-mediated signal activation and showed the highest pathogenicity. These findings strongly suggest that RABV N protein plays an important role in escaping the innate immune response in the brain by promoting effective viral dissemination. In addition, several studies have shown that in the early stages of RABV infection, the N protein of the CVS strain co-located with the Bax protein (a member of Bcl-2 family) in the mitochondria of infected cells to prevent its activation [22]. This effectively inhibited the caspase-dependent apoptosis of the mitochondria, which in turn provided additional time for virus replication and transcription [19]. However, the expression of N protein had no effect on the expression level of Bcl-2 family members [22]. Therefore, we suggest that N protein may play an important role in the host innate immune response by avoiding the production of IFN and inhibiting apoptosis.

### 2.2. The Pathogenic Mechanisms of RABV P Protein

The P protein is a multifunctional protein, consisting of a long-disordered N-terminal domain (amino acid 1–90), a central dimerization domain (DD, amino acid 91–133), a disordered linker (amino acid 134–185) and a C-terminal domain (CTD, amino acid 185–297), containing a large number of host as well as viral protein binding sites [23]. Amino acids at positions 1–60 of the P protein bind to the N protein, which prevents the N protein from binding to host nonspecific RNA [24], and amino acids at positions 11–50 of the P protein activate the L protein [25,26]. The transcription and replication of RABV are dependent on the binding of the PCTD to the N/RNA complex [27]. The P protein participates in viral RNA synthesis as a co-factor of the RNA-dependent RNA polymerase (RdRp). The P protein provides a potential regulatory center in replication and immune evasion, not only playing a key role in replication, but also escaping innate immune responses by binding to STAT transcription factors [28]. These interactions are mediated by the PCTD. Cell-based analysis showed that STAT1 and the N protein did not compete for the P protein. Thus, interactions that appear to be essential to replication and immune evasion can occur simultaneously, so that P protein binding to activated STAT1 can potentially occur without interrupting the interaction involved in replication. These data suggest that replicates may be directly involved in STAT1 antagonism. Three regions of PCTD region A (Ile201-Phe209), region B (Asp235-Lys237), and region C (Leu276-Val277) form an extended interface combining STAT1 [29]. In addition, the P protein plays a vital role as a type I IFN antagonist by inhibiting the phosphorylation of IFN regulatory factor 3 (IRF 3) [30,31]. Moreover, it inhibits the IFN-mediated JAK/STAT signal transduction [32,33,34]. Yamaoka et al. [35,36] found that the P deficient strain of Ni-CE replicated in muscle cells less efficiently than the parental strain Nishigahara. The P-deficient strain Ni-CE was defective in viral IFN antagonists in muscle cells. However, treatment with IFN-β neutralizing antibody improved the replication efficiency of Ni-CE in muscle cells, highlighting the importance of IFN antagonism. These results suggest that the P protein plays a key role in evading innate immune responses, rendering it a potential therapeutic target for RABV post-exposure prophylaxis (PEP) or anti-viral drugs. In addition, the interaction of the host ribosomal large subunit protein L9 (RPL9) with RABV P protein was screened through phage display technology [37]. During RABV infection, the P protein binds to L9, which translocates from the nucleus to the cytoplasm, inhibiting the initial stage of RABV transcription. However, it is unclear whether the interaction between the P protein and RPL9 affects the binding affinity with genomic RNA [38]. In addition, the P protein can also interact with the cytoplasmic dynein light chain LC8, which not only promotes the transcription of viral RNA, but also assists with the axonal transportation of virus within neurons [3,39]. In addition, the PCTD amino acids 106–131 can interact with the focal adhesion kinase to regulate RABV infection [37].

### 2.3. The Pathogenic Mechanisms of RABV M Protein

The M protein plays a crucial role in virus assembly, morphogenesis and budding during RABV replication as a pathogenicity determinant [40,41]. Amino acid at position 95 of the M protein is a cytopathic determinant of RABV and has been shown to play an important role in cell membrane disruption, but not in phosphatidylserine (PS) exposure [42,43]. The M protein interacts with the RNP and the G protein, participating in the recruitment of RNP to the host cell membrane and the budding of encapsulated viral particles even in the absence of G protein [13,36,44]. The M protein can also regulate the JAK-STAT pathway in coordination with the P protein. In unstimulated cells, both the M and P proteins were found to interact with JAK1. After stimulation by type I IFN, the M protein switches toward a pSTAT1 interaction. This leads to an enhanced ability of the P protein to interact with pSTAT1 and restrain it in the cytoplasm, thus inhibiting the JAK-STAT signaling pathways. In addition, the amino acids 77, 100, 104, and 110 of the M protein also play a role in inhibiting the JAK-STAT pathway [45]. The M protein affects pathogenicity by regulating viral replication and facilitating cell-to-cell spread [46]. Finke et al. [47] found that the M protein regulated RNA synthesis and affected the balance of replication and transcription products. The M protein can be used as an alteration regulatory switch to raise the initial mRNA synthesis level, resulting in a changing of RNA synthesis to facilitate the viral assembly of genomic RNA. Zan et al. [48] found that the overexpression of M protein can increase the expression of histone deacetylase 6 (HDAC6), which increases the transcription and replication of RABV through microtubule depolymerization. Zan et al. [22] observed that the M protein partially acts on the mitochondria through caspase-dependent or independent pathways in the late stage of infection, inducing mitochondrial apoptosis, thus promoting viral replication and transmission. Ben Khalifa et al. [49] reported that the M protein interacted with the C-terminal specific region of RelAp43, one of the six members of the NF-κB family, inhibiting NF-κB signal transduction and IFN-β transcription. Although the M protein is the smallest structural protein, it plays a unique role in viral assembly, regulating viral RNA synthesis, balancing replication and transcription products, as well as in evading innate immunity.

### 2.4. The Pathogeni Mechanisms of RABV G Protein

**Figure 2:**
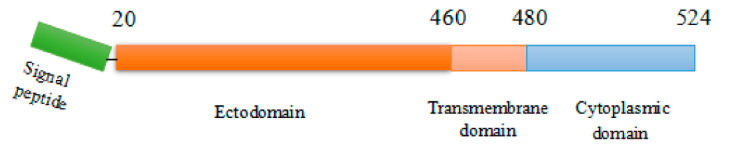
Domain structure of full-length G protein.

The G protein is a type I glycosylated protein, composed of 524 amino acids. During G protein maturation, the signal peptide is removed, and the mature G protein contains 505 amino acids [49]. A mature G protein can be divided into three parts (Figure 2): the extracellular domain; the transmembrane domain; and the cytoplasmic domain. The extracellular domain exists in the form of a homotrimer, each monomer containing 439 amino acid residues. The transmembrane domain comprises around 20 amino acid residues (aa 460 to aa 480). The cytoplasmic domain encompasses 44 amino acids in the inner membrane that extends into the cytoplasm of infected cells and interacts with M proteins to complete viral assembly [5,50].

As the main antigen inducing host VNA activity, the G protein can induce humoral immunity and stimulate T cells, which, if inhibited, may be related directly to viral virulence [50]. The G protein is involved in neurotropism [4,51,52] through the recognition of and adhesion to cell receptors, such as heparin sulfate [53], the acetylcholine receptor (nAChR) [54], the neural cell adhesion molecule (NCAM) [55] or the low-affinity neurotrophic receptor (p75NTR) [56], and the metabolic glutamine receptor subtype II (mGluR2) [57], which can be selected as targets for disease mediation.

Besides VNA induction, the G protein is also a determinant in activating host innate immune responses [51]. Zhang et al. discovered that by exchanging G proteins between street and fixed RABV strains, the laboratory strain elicited stronger innate immune and inflammatory responses than the street virus or recombinant virus expressing street virus G protein [51]. These results confirmed that laboratory strains induced disease through immune-mediated processes, while street virus or recombinant virus expressing street virus G protein induce disease through mechanisms other than immune-mediated pathogenesis. Réza Etessami et al. [58] successfully rescued G gene-deleted virus SAD delta G, which could not be transmitted between cells and showed no pathological changes in mice. In the context of the genome of RC-HL, Ito et al. [59] rescued a chimeric virus with the G gene of the Nishigahara strain, which caused death in adult mice compared with the more attenuated viral strain RC-HL. These results showed that chimeric virus containing the G protein of the Nishigahara strain was pathogenic to adult mice. Takayama-Ito et al. [60] mutated the 333 site amino acid of G protein from Glu to Arg, which restored the pathogenicity of the attenuated strain HEP-Flury, indicating that the 333rd amino acid of G protein played a role in pathogenicity in adult mice. Certain amino acids of G protein are believed to affect the pathogenicity of RABV. Takayama-Ito et al. [61] replaced the amino acids of the RC-HL strain with those of Nishigahara strain at positions 242, 255, and 268 of the G protein. The RC-HL strain showed the same pathogenicity as the Nishigahara strain, and the results showed that at least three amino acids were associated with enhanced pathogenicity. The D255G mutation of the G protein decreased viral neurotropism and significantly decreased replication in the brains of mice. In addition, the D255G mutation of the G protein enhanced the immune response of mice, which helped to clear RABV after infection, and the D255G mutation was genetically stable in vitro and in vivo [62]. Li et al. [63] found that a K83R mutation of the G protein of the RABV SAD strain enhanced the blood-brain-barrier (BBB) permeability and further reduced the pathogenicity of the SAD strain. These results suggest that the Arg83 and Lys83 mutations reduce pathogenicity and affect BBB permeability, both of which are key factors in preventing RABV infection. The Gly349Glu of the G mutation reduced RABV pathogenicity through enhanced immune response and increased BBB permeability. This study provided a new reference site, G349, which attenuated RABV pathogenicity [64]. The character of the C terminal of RABV with the PDZ binding motif (PBM) regulated viral virulence. The neural protein partners recruited by PBM may change the function of host cells. Ghassemi et al. [65] believed that the expression of RABV G promoted both short-and long-term synaptic plasticity in the hippocampal dentate gyrus (DG), suggesting that it may involve presynaptic and postsynaptic mechanisms to alter synaptic function.

### 2.5. The Pathogenic Mechanisms of RABV L Protein

The L protein is a multifunctional protein, which plays an important role in viral replication and transcription. However, there are few studies on the function of L protein. The L protein, together with the P protein, forms the polymerase complex and the viral ribonucleoprotein RNP complex, along with the N and P proteins, to function as a viral RdRp and ultimately control RABV replication and transcription [66,67]. The L and P proteins bind to the dynein light chain 1 (DLC1), which influences microtubule organization and mediates cytoskeleton reorganization, to facilitate viral components’ transport within cells for primary transcription [68]. Tian et al. [69] mutated four key amino acid sites, K (1685), D (1797), K (1829), and E (1867) in the conserved tetramer region, based on vesicular stomatitis virus (VSV). In VSV, the L protein contains a conserved catalytic tetramer region K-D-K-E, which functions mainly as an N-7 and 2′-O-methyltransferase (MTase) in the process of viral mRNA capping. They constructed a series of recombinant rabies virus (rRABV) and showed that K1685 and K1829 of the L protein are more sensitive to the inhibition of type I interferon expression, which plays an important role in RABV pathogenicity by escaping innate immunity in vitro and in vivo [69].

**Figure 3:**
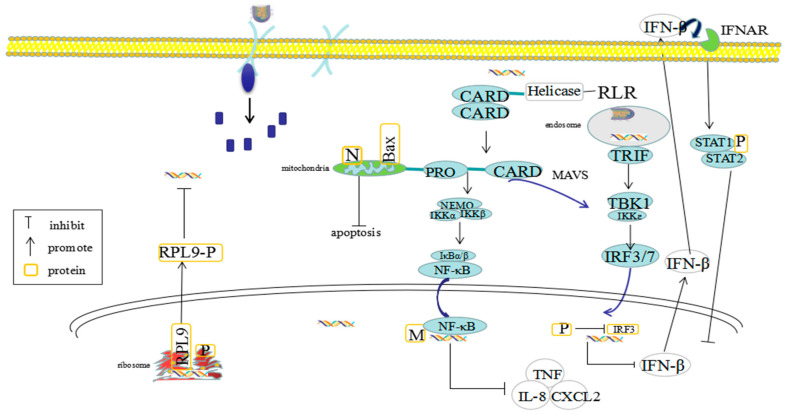
Schematic representation showing the interaction between rabies virus (RABV) viral proteins and host proteins in the innate immune system. N protein and activated Bax protein (a member of Bcl-2 family) co-located in the mitochondria of infected cells, and prevented the activation of Bax. In the early stage of infection, N protein effectively inhibited the apoptosis of mitochondria, prevented caspase-dependent and caspase-independent apoptosis, and provided time for virus replication and transcription. However, the expression of N protein had no effect on the expression level of Bcl-2 family members P protein plays the role of type I interferon (IFN) antagonist by reducing the phosphorylation of IFN regulatory factor-3 (IRF-3), inhibiting IFN production and blocking JAK/STAT signal transduction mediated by IFN. It plays an important role in virus escape from host innate immunity. P protein can bind to RPL9 protein directly in vivo and abroad, and transfer RPL9 protein from nucleus to cytoplasm, thus inhibiting the initial stage of RABV transcription. In the late stage of infection, M protein partially acts on mitochondria through caspase-dependent and caspase-independent pathways to induce mitochondrial apoptosis and promote virus replication and transmission. The interaction of M protein with C-terminal specific region of RelAP43, one of six members of NF-κB family, can inhibit NF-κB signaling and IFN- β transcription, which provides a way to avoid antiviral natural immunity. G protein can bind to cell receptor and mediate the invasion of virus to host, so G protein is closely related to the pathogenicity of virus.

Clearly, the RABV structural proteins play critical roles in viral binding, replication, and transcription, and also contribute to the evasion of host immune responses. Hence, their genetic manipulation can mediate viral pathogenicity in the host (Table 1; Figure 3).

## 3. Research on the Manipulation of RABV by Using Reverse Genetics

In 1885, Louis Pasteur and colleagues successfully created the first rabies vaccine for human use. For more than 100 years after Pasteur’s original work on rabies, vaccines have been improved continuously, from their propagation in nerve tissue to tissue culture. The large-scale production in Vero cells begun during 1985 is still widely used today (Table 2). Reverse genetic manipulation technology is a new method to study the function of specific genes by directly manipulating gene sequences and analyzing their phenotypic effects. It provides a powerful tool for molecular investigations of RNA viruses and is used broadly in RABV research. This technology laid the foundations for the application of genetically modified RABVs as viral vectors, expressing the exogenous genes of multiple antigens for consideration as vaccines, as well as for use in gene therapy.

### 3.1. The Establishment of Reverse Genetic Manipulation of RABV

Schnell et al. demonstrated that RABV may be generated by cDNA. They rescued an attenuated RABV strain, SAD B19, through reverse genetic technology [70]. Notable improvements included the use of hammerhead ribozymes (HHrz) to generate an exact 5′-end of the antigenomic RNA and the use of a CMV promoter to drive the expression of the antigenomic RNA [71,72]. Since then, a large number of studies have used reverse genetic manipulation technology to study the molecular biology of RABV and other negative-stranded RNA viruses. For example, Iton et al. rescued the RC-HL RABV, Japan’s avirulent strain used to produce inactivated rabies vaccine [59] in 2001, and Inouek et al. rescued the HEP-Flury strain in 2003 [71].

Reverse genetic manipulation technology has continued to improve since its inception. Initially, recombinant vaccinia virus was required to provide the T7 RNA polymerase. Thereafter, the system only required cells that could express the T7 RNA polymerase without the vaccinia virus. Additionally, the system was improved to use RNA polymerase II from a variety of cell lines, instead of the T7 RNA polymerase. Thus, the original low efficiency and cell-type-limited RABV rescue platform has evolved into a highly efficient system, which can now be applied to a variety of cell lines [73]. Rescuing the recombinant RABV from the cloned cDNA is still an inefficient process, as it relies on the formation of functional RNP complexes in the cells. These complexes are derived from RNA viral-like antigenome RNAs and three helper proteins. Ghanem et al. [74] optimized the use of the HHrz in 2012, not only increasing the rescue efficiency of RABV by more than 100-fold, but also significantly enhancing the reporter gene expression of the transfected small genome cDNA. The development of reverse genetic manipulation technology not only dramatically changed and broadened the field of the molecular biology of single-stranded, negative sense RNA viruses, but also opened a new avenue for studying RABV pathogenicity, as well as for developing novel RABV vaccines, and a next generation of RABV-based vaccine vectors.

### 3.2. The Application of Reverse Genetic Manipulation of RABV

#### 3.2.1. The Development of Attenuated Rabies Vaccines

Compared with traditional inactivated biologics, a recombinant RABV with replication defects is considered a safer and more effective vaccine candidate, which is expected to play a broader role in the prevention, control, and elimination of canine rabies. Viruses with gene deletions have special defects across their viral genome that may affect replication and the assembly of virus particles. They can only reproduce in complementary cell lines expressing deleted the gene products [8]. One of the first conditions for the rational application of a gene deletion strategy was for the appropriate complementary system to provide sufficient viral proteins without causing disease [8]. Among the five genes encoded by RABV, the deletion of the P and M genes offers the best potential for vaccine development, which can hinder the generation of viral offspring. Morimoto et al. [75] rescued a RABV with a P gene deletion from HEP-Flury (HEP), which can replicate and produce viral progeny in cell lines expressing the P protein. The def-p virus has no pathogenicity in adult mice, even if inoculated in the mouse brain. Meanwhile, the def-p virus induce higher titers of VNA and protected mice from the lethal infection of the CVS strain. McGettigan et al. [76] constructed a replication-deficient RABV vaccine, in which the M gene was deleted (RABV-⊿M), and no systemic or local reaction was found in dogs after vaccination, while rapid and effective VNA were induced. As for the remaining genes, their deletion either affects the immunogenicity of the virus or cannot be easily rescued, so their deletion is not suitable for vaccine production [75]. Therefore, RABV constructs with P or M gene deletions are expected to become safer alternatives to produce attenuated RABV vaccines by using reverse genetic technology. In addition, RABV vectored vaccines containing deleted G proteins can also include complementary modified G proteins, such as the 333 arginine-to-lysine site, which is the virulence-determinant site of RABV, and can be used for the production of attenuated strains. Targeted mutations in the G protein of RABV are the main approach for the construction of live attenuated RABV vaccines [77].

In addition to site mutations or the deletion of viral genes, the insertion of pro-apoptotic genes and/or an antiviral gene, the expression of inflammatory cytokines, and the addition of other viral genes [78] could be complementary strategies for the development of novel vaccine candidates. Réza Etessami et al. [58] showed that RABV containing two copies of the G gene increased protein expression. Faber et al. [79] found that the overexpression of the recombinant virus SPBNGA-GA G protein led to cellular apoptosis and enhanced antiviral immune responses. The improvement of immunogenicity and the loss of pathogenicity make SPBNGA-GA an excellent candidate strain for a modified live vaccine. Tao et al. [80] established the reverse genetic system for the LEP strain and produced recombinant LEP virus (rLEP-G) carrying two identical G genes. The VNA titer of the inactivated vaccine produced by rLEP-G in mice and dogs was significantly higher than that of the LEP-derived vaccine. These results showed that rLEP-G was a seed candidate strain for modified inactivated rabies vaccine. Increasing the expression level of the G protein in vaccine strains can not only significantly reduce the possibility of pathogenicity, but also greatly improve the production capacity and biosafety, as well as greatly reducing the production cost. These are key points in the modern production of RABV, especially the effective, safe and affordable vaccines urgently needed by developing countries. Therefore, recombinant RABV with a high expression of G protein may be an effective strategy for the development of novel rabies vaccines. Zhao et al. [5] used a new strategy to replace the RABV G protein signal peptide with the human IgG heavy chain signal peptide. The expression level of recombinant RABV G protein in the supernatant of HEK-293F cells increased more than 1000-fold. The rapid increase in RABV G protein expression was also observed in CHO cells. Thus, this approach could contribute to the development of a subunit rabies vaccine. Moreover, Zhang et al. [81] replaced the structural gene of the Venezuelan equine encephalitis virus (VEEV) with RABV-G and developed a new infectious propagation replicon particle (PRP), VEEV-RABV-G. The RABV G protein can effectively package chimeric replicon RNA into infectious particles, self-propagate in cell culture, and provide a high titer replicon. The VEEV-RABV-G particles were highly attenuated in adult mice and induced effective humoral immune responses, even at relatively low doses. Mice injected intramuscularly with VEEV-RABV-G vaccine avoided lethal RABV challenge by the intracranial (i.c.) route. Its safety and immunogenicity make VEEV-RABV-G a promising candidate strain for a safe attenuated live vaccine. Park et al. [82] developed a new type of RABV vaccine based on VSV, in which the VSV G protein was replaced by RABV G protein (VSV/RABV-GP). The titer of IgM after immunization was higher than the inactivated RABV, supporting its potential utility for vaccine production.

Fu et al. [83,84] found that recombinant RABV expressing-chemokines and cytokines (including GM-CSF) increase immunogenicity by inducing innate immunity and recruiting and activating dendritic cells and B cells. Recombinant RABV-expressing chemokines/cytokines can effectively prevent the occurrence of rabies in mice, indicating that recombinant RABV-expressing GM-CSF or flagellin is an effective intramuscular and oral vaccine candidate. In addition, recombinant RABV-expressing optimized high G mobility group Box 1 (HMGB 1) [85], DC binding peptide (DCBp) [86], and Fms-like tyrosine kinase3 ligand (Flt 3L) [87] enhanced humoral immunity by activating or promoting the maturation of host antigen presenting cells. The recombinant RABV-expressing CGXGC Motif Chemokine (CXCL 13) [88] can activate the Germinal Center (GC), which is the main site of antibody production, and promote the generation of GC B cells, thus continuously producing high levels of VNA. Other recombinant RABV-expressing interleukins, such as IL 6 [89], IL 7 [90], IL 15 [91], IL 18 [92], and IL 21 [93], could enhance humoral immunity and significantly increase VNA production. Among these, IL 7 could promote the production of memory B cells and enhance the long-term immune response. Recently, a recombinant RABV containing two copies of the codon optimization G gene was constructed. These results showed that the virulence of the recombinant RABV was attenuated, and immunogenicity was enhanced after G protein codon optimization, increasing expectation as a candidate for a next generation vaccine [94].

In addition to the studies mentioned above, some rRABV vaccines have already been licensed and used in the immunization of dogs and wildlife animals in the field, including ERA 333 [95], SPBN GAS [96], SPBN GASGAS [97], SAD dIND [32], ORA-DPC [98,99], rLBNSE-DCBp [86], and LBNSE-CXCL13 [88]; only ERA 333 and SPBN GASGAS have been successfully tested in wildlife target species [100]. As a result, the oral RABV vaccine strain SPBN GASGAS has successfully eliminated Sylvatic Rabies in foxes in Slovenia [101].

#### 3.2.2. RABV-Based Vectors as Vaccines against Other Infectious Diseases

There are several advantages to using RABV as a suitable expression vector: (i) the genome contains five genes with short transcription stop/start sequences flanking the genes, making it readily amenable to manipulation [102]; (ii) the replication cycle is exclusively in the cytoplasm, without the risk of recombination, reversion or integration to the host genome [9,70]; (iii) the stable expression of large and multiple foreign genes has been shown, up to 6.5 kb [9]; (iv) the resulting constructs induce a protective immune response in a broad variety of animals (Table 3).

Chi et al. [105] constructed a recombinant RABV containing the S1 protein of Middle East Respiratory Syndrome Coronavirus (MERS-CoV), based on the reverse genetic operation system of the SRV9 strain in BSR cells. The recombinant virus rSRV9-MERS S1 featured stable heredity and a high growth rate. RABV can be also used as a potential vector for HIV-1 and other diseases [109]. McKenna et al. [103] found that recombinant RABV-expressing HIV-1 antigen can induce strong and lasting cellular response to the HIV-1 antigen in mice, but the humoral response was not obvious. McGettigan et al. [110] discovered that recombinant RABV co-expressing HIV-1 protein and IL-2 can enhance the specific humoral immune response of HIV-1 antigen. Papaneri et al. constructed a recombinant RABV whose RABV G protein was replaced by the EBOV G protein. The results showed that it was safe and immunogenic in non-human primates [106,111]. Later, Keshwara et al. [112] further developed an effective EBOV vaccine based on the reverse genetic system rRABV vector, inserting the EBOV GP between the full-length N and P genes of the rRABV, to ensure a high level of transcription of GP (called RABV-ZGP). The RABV-G remained normal and could express both EBOV-GP and RABV-G in mammalian cells. The recombinant live virus RABV-ZGP was apathogenic in adult mice and was attenuated after intracranial injection in suckling mice, indicating the safety of that RABV vector. Kurup et al. [10] used a rRABV vector from the attenuated RABV strain SAD B19 and a recombinant VSV vector to develop a vaccine platform for henipaviruses. Tian et al. [104] used reverse genetics to prepare a recombinant RABV rLBNSE-Gn expressing severe fever with thrombocytopenia syndrome virus (SFTSV) glycoprotein (Gn). The addition of this Gn gene did not affect the replication of recombinant virus rLBNSE-Gn in NA and BHK-21 cells as compared with the parent rLBNSE strain. Intramuscular administration can induce a rapid and strong humoral response to RABV and SFTSV and provides a protective immune response in mice. Therefore, rLBNSE-Gn may be a promising bivalent vaccine candidate for the prevention of SFTS and rabies. Jin et al. [107] obtained a recombinant RABV-expressing Zika virus (ZIKV)-prM-E and evaluated the immunogenicity in BALB/C mice. The recombinant virus induced VNA against RABV and ZIKV and induced a specific cellular immune response, with the potential to prevent ZIKV and RABV infection. Zheng et al. [108] constructed a recombinant RABV expressing bovine ephemeral fever virus (BEFV) glycoprotein (LBNBG) using a reverse genetic system based on the recombinant strain LBNSE. The strong VNA against BEFV and RABV was elicited by immunizing mice with LBNSE-BG, which demonstrated complete protection against the lethal challenge of RABV. Moreover, more dendritic cells, B cells, and T cells were activated by LBNBG in immunized mice than those by LBNSE. These results showed that recombinant LBNSE-BG might be an economical and effective bivalent vaccine in BEF and rabies endemic areas.

## 4. Summary

In short, with the advancements in biology, RABV reverse genetic technology has become more efficient. Such progress has allowed the opportunity to use RABV as a vector to develop new vaccines, including against other major infectious diseases. However, major concerns remain about the relative safety of the vector used, so strict clinical evaluation is needed. With further advances, new RABV-based vaccines are expected to enter the market in the near future. RABV proteins interact with host proteins through a variety of ways to facilitate viral replication. Both direct and indirect mechanisms block innate immune effectors, which in turn hinder host defense pathways, enhancing viral replication. However, other specific mechanisms underlying the interaction between RABV and host cells require further investigation. For example, the post-translational modification of viral proteins and the role of acetylation modification during replication require elucidation. Given the utility of RABV reverse genetic systems, the study of the acetylation modification between virus and host can be further resolved for additional progress in the prevention, control, and treatment of this zoonosis.

## Figures and Tables

**Table 1:** Virus–Host Interactions in Rabies Virus Infection.

Structural Protein	Function
N (450aa)	(1) assembling viral RNA, forming RNP together with L and P
	(2) protecting viral genomic RNA from the cleavage of host cell nuclease
	(3) co-localization of Bax protein effectively inhibits mitochondrial apoptosis in the early stage of infection, prevents caspase-dependent and non-dependent apoptosis, and provides time for virus replication and transcription
	(4) inhibits the activation of RIG-Ⅰ and the expression of interferon regulatory factor 3 (IRF) and downstream antiviral genes
P (297aa)	(1) interacts with L, forms an active RNA polymerase, regulates the replication and packaging of virus RNA
	(2) interacts with LC8 not only promotes the transcription of viral RNA, but also facilitates the axonal transport of virus in neurons
	(3) interaction with L9 protein can inhibit the transcription and replication of virus
	(4) combined with BECN1, induces incomplete autophagy to destroy the host immune system and promote the replication of viral genome
	(5) interacts with FAK to regulate RABV infection
	(6) weakening the phosphorylation of IRF-3 to inhibit IFN production and block IFN mediated JAK/STAT signal transduction, play the role of type I interferon (IFN) antagonist, helping the virus to proliferate in host cells
M (202aa)	(1) tight connection with RNP, helps G protein to complete the budding of RABV virus particles
	(2) increases the expression of histone deacetylase 6 (HDAC6)
	(3) acts with P65 / Rel A to inhibit the response of NF-κB to IFN-β and block the replication of viral RNA
	(4) binds to the C-terminal domain of P43 / Rel A subunit in NF-κB reaction, resulting in the inhibition of NF-κB dependent gene regulatory factors
G (505aa)	(1) binds to the specific receptor (e.g., heparan sulfate, acetylcholine receptor (nAChR), nerve cell adhesion molecule (NCAM) or low affinity neurotrophic receptor p75NTR, metabotropic glutamate receptor subtype II (mGluR2)) on the cell
	(2) mediates the endocytosis of the virus into the cell
	induces the virus to produce neutralizing antibody, and determines the neurophagocytic property of the virus
L (2130aa)	(1) together with P protein, is responsible for viral genome replication, transcription and post-transcriptional processing
	(2) influences microtubule organization of and mediates cytoskeleton reorganization;
	contains a conserved catalytic tetramer region of K-D-K-E, which mainly performs the function of N-7 and 2′-O methyltransferase (MTase) during viral mRNA capping
	(4) the important factor in pathogenicity
	(5) escapes the innate immunity of host

**Table 2:** Development of RABV vaccine.

Years	Type of Vaccine
1885	Pasteur seedlings
1911	Sheep brain vaccine
1955	Suckling rat brain vaccine
1956	Duck embryo vaccine
1960	Primary hamster kidney vaccine
1965	Human diploid seedlings
1985	Vero cell vaccine

**Table 3:** RABV-Based Vectors as Vaccines Against Other Infectious Diseases.

Characteristics	Virus	Reason
(1) RABV genome contains five genes and with short transcription stop/start sequences flanking the genes	Human immunodeficiency virus-1 (HIV-1)	an intracellular life cycle and ability to stably express foreign antigens [103].
	Hepatitis C virus (HCV)	induce both a humoral and cellular response [10].
(2) The life cycle of RABV is exclusively in cytoplasmic, no recombination, reversion or integration observed	Severe Fever with Thrombocytopenia Syndrome virus (SFTSV)	induce high neutralizing antibodies in mice [104].
	Middle East respiratory syndrome coronavirus (MERS-CoV)	stable heredity and high growth titer [105].
(3) Stable expression of large and multiple foreign genes of up to 6.5 kb	Ebola virus (EBOV)	safe and immunogenic to non-human primates [106].
(4) RABV can induce a protective immune response in a variety of animals	Henipaviruses (HeV)	a killed RABV vaccine would be highly effective against HeV infections [9].
	Zika virus (ZIKV)	induced VNA against RABV and ZIKV and induced a specific cellular immune response, with the potential to prevent ZIKV and RABV infection. [107].
	Bovine Ephemeral fever virus (BEFV)	induce high neutralizing antibody against RABV and high specific antibodies against BEFV [108].

## Data Availability

Not applicable.

## Abbreviations

BBB	blood-brain-barrier
BEFV	bovine ephemeral fever virus
CD	cytoplasmic domain
CXCL	CGXGC Motif Chemokine 13
DCBp	DC binding peptide
DG	dentate gyrus
DLC1	dynein light chain 1
Flt 3L	Fms-like tyrosine kinase3 ligand
G	glycoprotein
GC	Germinal Center
HDAC6	histone deacetylase 6
HHrz	hammerhead ribozymes
HMGB 1	high G mobility group Box 1
IRF3	IFN regulatory factor 3
L	large RNA polymerase protein
M	matrix protein
MERS-CoV	Middle East Respiratory Syndrome coronavirus
MGluR2	the metabolic glutamine receptor subtype II
N	nucleoprotein
nAChR	the acetylcholine receptor
NC	nucleocapsid
NCAM	the neural cell adhesion molecule
P	phosphoprotein
p75NTR	the low affinity neurotrophic receptor
PBM	PDZ binding motif
PCTD	the C-terminal domain of P protein
PEP	post-exposure prophylaxis
PRP	propagation replicon particle
PS	phosphatidylserine
RABV	rabies virus
RdRp	RNA-dependent RNA polymerase
RNP	ribonucleoprotein
RPL9	ribosomal large subunit protein L9
rRABV	recombinant rabies virus
SFTSV	severe fever with thrombocytopenia syndrome virus
VEEV	Venezuelan equine encephalitis virus
VNA	virus-neutralizing antibodies
VSV	vesicular stomatitis virus
ZIKV	Zika virus
